# The Effect of Contact Investigations and Public Health Interventions in the Control and Prevention of Measles Transmission: A Simulation Study

**DOI:** 10.1371/journal.pone.0167160

**Published:** 2016-12-12

**Authors:** Wayne T. A. Enanoria, Fengchen Liu, Jennifer Zipprich, Kathleen Harriman, Sarah Ackley, Seth Blumberg, Lee Worden, Travis C. Porco

**Affiliations:** 1 Department of Epidemiology & Biostatistics, University of California at San Francisco, San Francisco, California, United States of America; 2 Francis I. Proctor Foundation for Research in Ophthalmology, University of California at San Francisco, San Francisco, California, United States of America; 3 Immunization Branch, Division of Communicable Disease Control, California Department of Public Health, Richmond, California, United States of America; 4 Department of Ophthalmology, University of California at San Francisco, San Francisco, California, United States of America; University of Waterloo, CANADA

## Abstract

**Background:**

Measles cases continue to occur despite its elimination status in the United States. To control transmission, public health officials confirm the measles diagnosis, identify close contacts of infectious cases, deliver public health interventions (i.e., post-exposure prophylaxis) among those who are eligible, and follow-up with the close contacts to determine overall health outcomes. A stochastic network simulation of measles contact tracing was conducted using existing agent-based modeling software and a synthetic population with high levels of immunity in order to estimate the impact of different interventions in controlling measles transmission.

**Methods and Findings:**

The synthetic population was created to simulate California`s population in terms of population demographics, household, workplace, school, and neighborhood characteristics using California Department of Finance 2010 census data. Parameters for the model were obtained from a review of the literature, California measles case surveillance data, and expert opinion. Eight different scenarios defined by the use of three different public health interventions were evaluated: (a) post-exposure measles, mumps, and rubella (MMR) vaccine, (b) post-exposure immune globulin (IG), and (c) voluntary isolation and home quarantine in the presence or absence of public health response delays. Voluntary isolation and home quarantine coupled with one or two other interventions had the greatest reduction in the number of secondary cases infected by the index case and the probability of escape situations (i.e., the outbreak continues after 90 days).

**Conclusions:**

Interrupting contact patterns via voluntary isolation and home quarantine are particularly important in reducing the number of secondary cases infected by the index case and the probability of uncontrolled outbreaks.

## Introduction

Despite the elimination of endemic measles transmission in the United States [1–3], measles cases continue to occur from time to time, mostly due to importation. In 2014–2015, there was a large measles outbreak that was linked to one or more Disney theme parks [4, 5]. The outbreak consisted of 131 cases involving 14 local health jurisdictions in California as well as other U.S. states and countries.

Each identified case requires extensive follow-up to confirm the infection and identify others who may have been exposed while the case was infectious. Contact tracing, a method of eliciting close contacts of cases, is used to identify individuals who may have been exposed to the case and need appropriate medical follow-up. For measles, post-exposure prophylaxis (PEP) can reduce an exposed person’s likelihood of infection if taken within the appropriate time from exposure. For example, measles, mumps, and rubella (MMR) vaccine should be administered within 72 hours from the time of exposure in order to be effective as PEP, while immune globulin (IG) should be administered within six days of exposure [6]. In addition, public health authorities can also advise persons who have been exposed or symptomatic individuals to stay at home (voluntary quarantine and isolation) [7]. However, public health authorities cannot always administer PEP to a person who is eligible for PEP within the appropriate time frame.

Contact tracing has been used in the control of many infectious diseases, including tuberculosis [8], smallpox [9], sexually-transmitted diseases [10–12], and severe acute respiratory syndrome [13–16]. Mathematical epidemiology studies have expanded on the empirical work to show that contact tracing effectiveness depends on the pathogen and setting, the fraction of secondary cases caused by the index case that is identified through contact tracing [17], the basic reproduction number for the pathogen, the fraction of transmission occurring asymptomatically, and the network structure [18, 19]. However, contact tracing requires a considerable investment of time and public health resources, even when a small number of contacts are identified [20]. In 2004, the Iowa Department of Public Health spent 2,525 personnel hours and spent $142,452 in direct costs to contain one case of measles [21]. Given the amount of resources required for contact tracing, there is a need to estimate the contributions of contact tracing and public health interventions to the control and prevention of measles transmission.

Using an agent-based model with eight different scenarios defined by three public health interventions (MMR PEP, IG PEP, and voluntary isolation and quarantine), we estimated the effects of contact tracing and public health interventions on the number of persons infected by an index case and the probability of uncontrolled outbreaks. The use of an agent-based model enabled us to model complex processes involving infectious disease transmission and a public health response in a synthetic population of individuals in order to compare potential outcomes among different scenarios. As a sensitivity analysis, we examined the effects of two measles vaccination coverage levels among individuals 1 to 18 years of age: (1) 95% to 100% and (2) 85% to 95%.

## Methods

### Overview

We used an agent-based model to simulate measles outbreaks while incorporating key features of measles epidemiology. First, we assumed that imported measles cases occurred in a population with high population immunity. Second, we used established estimates for the incubation period and duration of infectivity, and assumed a high transmissibility. Third, we modeled a public health response to identified cases that involved case and contact investigations with reasonable delays. Fourth, we modeled the use of public health interventions (MMR PEP, IG PEP, and voluntary isolation and quarantine) [22]. Using a synthetic population that contained family structure, household size, age distribution, schools, workplaces, neighborhoods, and population immunity, measles outbreaks were simulated with one infected case introduced into a synthetic population each time. The agent-based model was implemented using the Framework for Reconstructing Epidemiological Dynamics, an open-source agent-based modeling system ([23] and http://fred.publichealth.pitt.edu). We describe the details of each of the features and their assumptions in the sections below.

### Synthetic population

The synthetic population mimicked a medium-sized county population living in California based on California Department of Finance 2010 census data (http://www.dof.ca.gov). The county was chosen such that the distribution of demographic characteristics (percent female, percent under five years old, percent under 18 years old, and percent 65 years and older) were within 10% of the state’s demographic distribution. Each individual in the synthetic population had demographic information (e.g., age and gender) and locations of social activity (i.e., household, school, daycare, neighborhood, and workplace). The household is an important setting to include because the household is a major site for transmission of respiratory pathogens [24]. Infectious disease transmission is known to occur at higher rates in schools because of the close and sustained contact between students [25–27]. In addition, we allowed for the possibility that transmission could occur in daycare settings by adding daycares to the synthetic population based on data from the California Department of Social Services’ Community Care License Division (http://www.ccld.ca.gov) [22]. During each simulated day, individuals interacted with other individuals who shared the same locations of social activity. The details of our model for individual interactions among the different locations of social activity has been described previously [22]. Further details about the synthetic population can be found online (http://www.epimodels.org/10_Midas_Docs/SynthPop/2010_synth_pop_ver1_quickstart.pdf).

### Population Immunity of the Synthetic Population

Children less than one year of age were assumed to have a 67% level of protection due to maternal antibodies [28]. We conducted two sets of simulations in which vaccine coverage ranged from 95% to 100% and 85% to 95% among individuals ages 1 year to 18 years of age; age-specific immunity levels were estimated based on measles vaccine coverage and vaccine efficacy. The average transmission chain size can increase even with small decreases in the overall level of population immunity [29]. To model clusters of susceptible children in a highly vaccinated population, the clustering of the vaccination status of individuals 1 to 18 years of age within households only was allowed to vary from 0 (random vaccination assignment) to 1 (completely positive assortative vaccine status); the details of clustering measurement were described previously [22]. Values of clustering between 0 and 1 represent intermediate vaccination configurations between the two extremes. The immunity levels for individuals greater than 18 years old were consistent with age-specific prevalence estimates of measles antibody in the United States [30].

### Modeling Measles Outbreaks

The age of the index case was sampled from the observed age distribution of measles cases in California from 2000–2012. A susceptible individual of this age was chosen from the synthetic population to represent the index case, the source of the imported measles virus into the synthetic population. We modeled the natural history of measles using an SEIR framework [31] where prodromal symptoms begin 8 to 14 days after exposure [32, 33]. The stochastic nature of the model permits simulation of the probability of no transmission as well as variability in the initial growth rate of the outbreak [34]. After the incubation period, infectious cases were able to transmit measles to different contacts in household, daycare, school, workplace, and neighborhood settings. We simulated transmission for 90 days after the introduction of measles into the synthetic population. Key parameters of our model are given in Table 1.

**Table 1:** Key parameters for the agent-based measles model.

Parameter Description	Range of Values	Reference
Vaccine coverage	[0.95 to 1.0] and [0.85–0.95]	[35, 36]
Clustering of vaccination status among children	0.0–1.0	Assumed
Household contact probability	0.01–1.0	
Neighborhood contact rate	0.5–7.0 contacts per day	
Workplace contact rate	0.0–7.0 contacts per day	Synthetic population
School contact rate	3.0–20.0 contacts per day	http://www.cde.ca.gov/ds/sd/dr/cefteachavgclssize.asp
Daycare contact rate	3–20	http://www.daycare.com/california
Household transmission probability per contact	0.9–1.0	[37]
Neighborhood transmission probability per contact	0.9–1.0	[37]
Workplace transmission probability per contact	0.9–1.0	[37]
School transmission probability per contact	0.9–1.0	[37]
Daycare transmission probability per contact	0.9–1.0	[37]
Trace probability	0.9–1.0	Assumed
Intervention delay 1	1.0–3.0 days	J. Zipprich, personal communication
Intervention delay 2	1.0–3.0 days	J. Zipprich, personal communication
Self-report delay	1.0–6.0 days	Assumed
Cooperation probability	0.9–1.0	Assumed
Contact finding probability	0.9–1.0	Assumed
MMR PEP efficacy (1 dose MMR)	0.92–0.95	[38, 39]
IG PEP efficacy	0.60–0.90	[40–42]
Home quarantine probability	0.90–1.0	Assumed
Home stay probability (the probability that someone who feels sick stays home)	0.0–1.0	Assumed
MMR vaccine efficacy (two doses)	0.99–1.0	[38, 39]

### Modeling Case/Contact Investigations and Public Health Interventions

In our simulation, the health department was notified of index cases three to six days after the onset of their symptoms. Then, health department staff followed up with identified cases via case investigations. Contact tracing identified household, daycare, school, and/or workplace setting contacts of index cases during their infectious period. In the model, if the identified case cooperated with a health department’s investigation, every contact exposed to that case in the previous seven days (including the day that case was identified) had a chance to be found. We assumed that contact investigations could not occur among neighborhood contacts because it is unlikely that neighborhood contacts could be identified.

**Fig 1:**
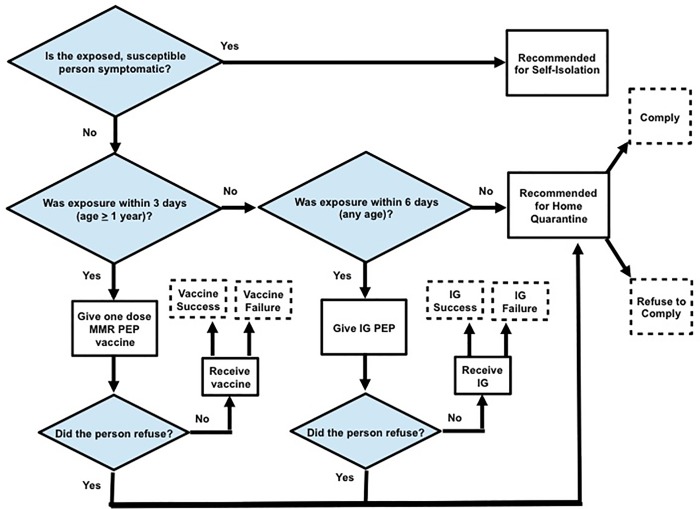
Public health intervention decisions for susceptible and exposed contacts. Abbreviations: MMR, measles, mumps, and rubella; IG, immune globulin; PEP, post-exposure prophylaxis.

To minimize the likelihood of developing measles after exposure, public health interventions were given to identified contacts as needed based on the exposure date determined for that contact. One dose of MMR PEP was given to exposed, susceptible individuals over 12 months of age within three days of exposure, if possible. In addition, immune globulin (IG) PEP was given to exposed, susceptible individuals of any age within six days of exposure, if possible, although current CDC recommendations now suggest restricting IGIM to individuals < 66 lbs. due to declines in the measles IgG level in the U.S. blood supply. Exposed, susceptible individuals who did not receive MMR PEP vaccine or IG PEP were asked to refrain from contact with non-household members (i.e., voluntary home quarantine) until the end of their incubation period. In the agent-based model, a person could refuse PEP or voluntary home quarantine recommendations. If an identified case refused all interventions, or all interventions given to that person failed (possible because of incomplete efficacies), that person could still transmit measles to others if infected. However, any newly-infected individuals would eventually visit a doctor in three to six days after the onset of his or her symptoms, and the health department would be notified and initiate a contact investigation based on the newly-identified individual. In practice, self-isolation is recommended if an exposed, susceptible person is symptomatic. A graphical representation of the public health intervention decisions for susceptible contacts is given in Fig 1.

### Main Outcomes

Model parameter values were selected using a Latin Hypercube sample [43, 44] of parameters chosen uniformly from the parameter ranges given in Table 1. Using the 1024 parameter sets derived in this way, measles epidemics were simulated over 90 simulated days to evaluate the effectiveness of contact tracing and public health interventions for all eight different scenarios defined by different combinations of MMR PEP vaccine, IG PEP, and voluntary isolation and home quarantine with public health response delays. The scenario without MMR PEP, IG PEP, and voluntary isolation and home quarantine served as the reference scenario to which the other seven scenarios were compared. For each scenario, we conducted 2^8^ = 256 replications in order to balance computing resource availability with having enough replications to identify the distributions of the main outcomes. Each replication corresponds to an introduction of a measles case and following the subsequent chain of transmission. In order to evaluate the effectiveness of contact investigations with public health interventions, we examined two outcomes in our simulations: (1) the number of individuals a randomly chosen index case infected (referred to as “R primary”), and (2) the proportion of the 256 iterations that resulted in at least one measles case (i.e., someone who was infectious) at the end of the 90 simulated days. We did not consider severity of measles infection (e.g., death and hospitalizations) as main outcomes in our comparisons. Ninety days was chosen as the end of simulation time because large measles outbreaks in the era of elimination have not exceeded 11 weeks in duration [45]. Thus, the simulation was repeated 256 times for each of the 1024 parameter sets under the eight scenarios to obtain a distribution for the main outcomes; the median and interquartile range of each outcome across the parameter sets for each of the eight scenarios is presented. The differences in outcomes were compared under different scenarios.

## Results

The median value of R primary, the average number of secondary cases caused by the index cases, among the simulations for each of the eight scenarios in a population with high levels of vaccination coverage (95% to 100%) among individuals 1 to 18 years of age are given in Table 2. The median value of R primary ranged from 0.56 in the scenario where all three interventions were given to 0.84 in the scenario where no interventions were given. The scenarios with voluntary home quarantine (i.e., scenarios 2, 6, 7, and 8) had a greater decrease in the median number of secondary cases in comparison with other scenarios that did not include voluntary home quarantine. The median probability of having active cases remaining after 90 days, across all parameter sets, ranged from 0.004 (for the scenario with all three interventions) to 0.09 (for the scenario without interventions), demonstrating that measles transmission can die out in the absence of any interventions given the high level of vaccination coverage in the synthetic population.

**Table 2:** Reduction in secondary cases caused by an index case and the probability of escape for the eight scenarios with public health response delays and 95% to 100% vaccination coverage among children 1 to 18 years of age^a^.

No.	Intervention Strategies			Number of Secondary Cases Caused by the Index Case		Reduction in the Median Number of Secondary Cases Caused by the Index Case	Probability of Escape^b^		Reduction in the Median Probability of Escape
	MMR PEP	IG PEP	Voluntary Isolation & Quarantine	Median	IQR		Median	IQR	
1	No	No	No	0.84	0.58, 1.42	0	0.09	0.01, 0.37	0
2	No	No	Yes	0.71	0.50, 1.05	-0.13	0.05	0.004, 0.21	-0.05
3	No	Yes	No	0.69	0.45, 1.20	-0.15	0.04	0, 0.25	-0.06
4	Yes	No	No	0.70	0.46, 1.23	-0.14	0.04	0, 0.26	-0.05
5	Yes	Yes	No	0.66	0.43, 1.16	-0.18	0.03	0, 0.23	-0.07
6	Yes	No	Yes	0.60	0.42, 0.90	-0.24	0.01	0, 0.10	-0.08
7	No	Yes	Yes	0.58	0.40, 0.88	-0.26	0.01	0, 0.08	-0.09
8	Yes	Yes	Yes	0.56	0.38, 0.85	-0.28	0.004	0, 0.06	-0.09

Abbreviations: PEP, postexposure prophylaxis; MMR, measles, mumps, and rubella; IG, immune globulin; IQR, interquartile range

^a^ The reduction of median outcomes may not equal the difference using the numbers displayed due to rounded values.

^b^ The probability of escape is defined as the proportion of 256 iterations in which there was at least 1 measles case at the end of 90 days of simulation.

### Sensitivity Analysis when Vaccination Coverage is 85% to 95% among Individuals 1 to 18 Years of Age

When measles vaccination coverage in the synthetic population was lowered to 85% to 95% among individuals 1 to 18 years of age, all median estimates for the two outcomes increased. The scenarios with voluntary home quarantine coupled with one or two other interventions (i.e., scenarios 6, 7, and 8) had the largest decrease in the median number of secondary cases in comparison with the other scenarios (Table 3). The median value of R primary ranged from 0.90 (scenario with all three interventions) to 1.52 (the scenario with no interventions). There was a reduction in the median probability of escape across all parameter sets when considering a scenario with no interventions with a scenario with all three interventions.

**Table 3:** Reduction in secondary cases caused by an index case and the probability of escape for the eight scenarios with public health response delays and 85% to 95% vaccination coverage among children 1 to 18 years of age^a^.

No.	Intervention Strategies			Number of Secondary Cases Caused by the Index Case		Reduction in the Median Number of Secondary Cases Caused by the Index Case	Probability of Escape^b^		Reduction in the Median Probability of Escape
	MMR PEP	IG PEP	Voluntary Isolation & Quarantine	Median	IQR		Median	IQR	
1	No	No	No	1.52	1, 2.47	0	0.43	0.24, 0.64	0
2	No	No	Yes	1.26	0.89, 1.81	-0.26	0.36	0.19, 0.54	-0.07
3	No	Yes	No	1.13	0.74, 1.90	-0.39	0.27	0.07, 0.54	-0.16
4	Yes	No	No	1.18	0.76, 1.97	-0.33	0.30	0.08, 0.56	-0.12
5	Yes	Yes	No	1.07	0.68, 1.82	-0.45	0.23	0.04, 0.51	-0.19
6	Yes	No	Yes	1.00	0.69, 1.46	-0.52	0.20	0.04, 0.41	-0.22
7	No	Yes	Yes	0.95	0.66, 1.41	-0.56	0.16	0.02, 0.37	-0.27
8	Yes	Yes	Yes	0.90	0.62, 1.35	-0.62	0.10	0.008, 0.31	-0.33

Abbreviations: PEP, postexposure prophylaxis; MMR, measles, mumps, and rubella; IG, immune globulin; IQR, interquartile range

^a^ The reduction of median outcomes may not equal the difference using the numbers displayed due to rounded values.

^b^ The probability of escape is defined as the proportion of 256 iterations in which there was at least 1 measles case at the end of 90 days of simulation.

## Discussion

By examining the scenario outcomes defined by different interventions aimed to prevent measles transmission using an agent-based model, we were able to demonstrate the effects of each control component of a network-based control strategy on the number of secondary cases caused by an index case and the likelihood of uncontrolled outbreaks in a population with a high level of immunity. Our definition of an uncontrolled outbreak is conservative in that we only considered infectious individuals at the end of 90 days. An exposed person could still go on to become a case beyond 90 days but we did not include exposed individuals in our determination of uncontrolled outbreaks. The simulation results showed that the most effective component in reducing measles transmission in this population is voluntary isolation and home quarantine, i.e., activities that affect contact patterns among individuals.

However, our simulation results are based on making specific assumptions about population immunity, as well as individual and public health response behaviors. First, the clustering of population immunity parameter only considered clustering among children less than 18 years of age; we did not consider clustering of immunity among adults. This may occur because parents make vaccination decisions for all of their children. Clustering of immunity can occur as a result of any opinion formation process [46] or personal beliefs [47] in which the degree of clustering of susceptibility may affect the likelihood of disease outbreaks [46] and infectious disease transmission dynamics [48]. In some instances, clustering may reduce the contact tracing efficacy [49]. We did not explore the consequences of different levels of clustering among adults on the overall effectiveness of different components of the response to control measles transmission, nor did we present the geographic spread of measles in the population as has been done previously [50]. Second, we did not explicitly consider measles transmission among healthcare settings. We limited our investigations to the household, workplace, school, and daycare settings. Although transmission could occur in the neighborhood settings, we did not enable contact tracing to occur in the neighborhood since it would be unlikely to identify the contacts in a neighborhood setting. Third, we obtained behavioral aspects of our model (individual behavior and public health response behavior) from the published literature and/or expert opinion. When input estimates were unavailable, we chose inputs that were plausible based on the overall goals of the study. For example, we wanted to estimate the effectiveness of home (volunteer) quarantine on the control of measles transmission. The level of compliance we assumed for home quarantine recommendations would affect our effectiveness estimates. It is likely that the true compliance would vary depending on perceived risks, the risks and benefits of potential consequences, and perceived difficulty with compliance as has been demonstrated previously with SARS [51]. In addition, recommendation refusals may also be correlated with one another. Individuals who refuse MMR PEP or IG PEP may also fail to comply with home quarantine recommendations. Furthermore, operational estimates for measles contact tracing are not available in the published literature; further research is urgently needed in order to refine these estimates.

Maintaining a high level of population immunity appears to be the best way to prevent measles [47]. Exposure to a measles case is not a contraindication for vaccination and MMR PEP can provide some protection if given within 72 hours of exposure [6]. If a person does not receive MMR PEP within 72 hours of exposure, IG PEP may prevent or modify measles in a susceptible person if given within six days. MMR PEP, IG PEP, isolation and quarantine can help lower the number of secondary cases caused by the index case and the chain size should measles outbreaks occur.

## Conclusions

In this agent-based, stochastic simulation study, we simulated measles outbreaks in a synthetic population with high immunity over a range of plausible transmission and intervention parameters in order to evaluate the effectiveness of components of the public health response to control and prevent measles transmission. The simulation results suggest that contact investigations and interventions lower the number of cases introduced by an index case into the population. Voluntary isolation and home quarantine are particularly important in reducing the number of secondary cases infected by the index case and the probability of uncontrolled outbreaks.

## Abbreviations

IG: immune globulin
MMR: measles, mumps, and rubella
PEP: post-exposure prophylaxis
SD: standard deviation
IQR: interquartile range
